# The Conversations of the Senior Editor, Etc.

**Published:** 1881-09-20

**Authors:** 


"*The Conversations*" of the Senior Editor which have been running for some time in our Journal will cease with the present number. They will be put into pamphlet form, and will contain a supplement on *What Constitutes the True Woman.* They were, of course, not intended as a standard work for the profession, but rather for non-professional readers, whom it was hoped would find in them good and useful suggestions upon a subject of great interest and importance, and upon which much ignorance prevails. It was believed also that the medical student, many of whom read the RECORD, and young practitioners at least would find interest and profit in "Conversations," which were based upon the actual experience and observation of the writer, and which contain many valuable hints in regard to the manner of approaching and talking to patrons and patients, together with practical suggestions which can be made available and useful in the social and professional walks of life. The pamphlet may be obtained by an order to A. M. Bergstrom, publisher, Atlanta. Price, 50 cents.

